# ‘As You Set Out for Ithaca’: VIEW- A Visual Tool for Teaching Ethical Decision Making in Medical Practice

**DOI:** 10.5334/pme.1543

**Published:** 2025-07-24

**Authors:** Dikla Agur Cohen, Liv Shadmi, Aya Biderman, Gila Yakov

**Affiliations:** 1 Department of Family Medicine, The Ruth and Baruch Rappaport Faculty of Medicine, Technion, Israel; 2 The Department of Family Medicine, The Ruth and Baruch Rappaport Faculty of Medicine, Technion, Israel; 3 Department of Family Medicine, Faculty of Health Sciences, Ben-Gurion University of the Negev, Israel; 4 The Max Stern Yezreel Valley College, Israel

## Abstract

**Background & Need for Innovation:**

Ethical dilemmas in healthcare often involve navigating emotional and value-laden challenges. Traditional teaching approaches, such as principle-based frameworks, highlight the theory but do not contain tools to integrate emotional reflection and value prioritization, leaving a gap in preparing physicians for real-world ethical decision making.

**Goal of Innovation:**

We introduce a novel visual tool named VIEW (**V**isual **I**nsight for **E**thical **W**isdom) that enhances ethical decision making by fostering reflection on values, emotions, and professional identity.

**Steps Taken for Development and Implementation:**

The VIEW tool was developed as an interactive framework that allows users to map ethical dilemmas visually. It was implemented in three workshops with 31 participants, including junior and senior physicians and medical educators. Participants used the VIEW to analyze real-life ethical challenges. They created 31 visual maps that captured emotional responses, core values, and interpretations of dilemmas. We qualitatively analyzed these maps, alongside other observations and interviews, to identify key themes.

**Outcomes of Innovation:**

Preliminary evaluations indicated that the VIEW tool effectively bridges theory and practice. It externalized thought processes, encouraging emotional awareness, and promoting value-based reasoning. Participants reported improved capacity to navigate complex dilemmas and deeper engagement with their professional identity.

**Critical Reflection on the Process:**

This visual tool integrates cognitive and emotional dimensions of ethical reasoning, addressing the limitations of traditional approaches. Its implementation highlights the importance of reflection in medical education and clinical practice. Further research is needed to explore the tool’s broader applicability.

## Background & Need for Innovation

‘*As you set out for Ithaka*,

*hope your road is a long one*,

*full of adventure, full of discovery*…’

C.P. Cavafy’s words [1] echo the journey of healthcare professionals who navigate the challenging landscape of medical ethics—wisdom, reflection, and growth. Medical ethics is central to shaping professional identity (PI) in healthcare. It integrates values, behaviors, and responsibilities [2], while reinforcing key principles such as patient-centered care, autonomy, and social justice into medical professional conduct [3]. Ethical education fosters reflection, empathy, and integrity. It helps professionals align personal and institutional values amidst clinical challenges. This process supports professionalism, fostering trust and collaboration [4,5]. According to Cruess et al., PI formation involves internalizing the values and norms of the medical profession, transforming individuals into healthcare professionals through reflective practices and ethical reasoning. This connection between reflective practice and PI formation is well-established in the literature [6,7]. Modern medical ethics have evolved from Percival’s work in 1803 through the post-World War II Nuremberg Code, culminating in today’s comprehensive ethical frameworks. These frameworks include the widely adopted four principles approach of autonomy, beneficence, nonmaleficence, and justice as well as MacIntyre’s revival of Aristotelian virtue ethics, which emphasizes virtues over rigid principles [8,9,10].

Despite this solid theoretical foundation, teaching ethical decision making remains challenging. Recent literature emphasizes context-specific approaches such as Kaldjian’s phronesis framework, which prioritizes contextual understanding and virtues [11]. Various teaching methods—case-based learning, small group discussions, workshops, reflective writing, and patient perspectives—are employed to develop practical wisdom and critical thinking skills [12].

The four topics approach offers a structured framework for analyzing ethical dilemmas in healthcare by focusing on medical indications, patient preferences, quality of life, and contextual features [13]. While this structured approach helps practitioners and trainees consider ethical dilemmas in a systematic manner, it may oversimplify complex issues and struggles to address emotional aspects or support rapid decision making in time-sensitive situations.

Current methods emphasize principle-based ethics, focusing less on the importance of virtues and personal values, which are crucial for holistic reasoning. A one-size-fits-all approach fails, however, to address variability in learner experiences and cultural contexts [14]. Furthermore, the increasing reliance on technology in medical practice creates gaps in training tools that emphasize human-centered, reflective processes [15]. The theoretical framework for the proposed visual tool is inspired by Visual Thinking Strategies (VTS), which integrate visual perception and analytical thinking to enhance clinical skills. The tool, which adopts VTS principles—structured observation, reflective analysis, and collaborative dialogue—aims to cultivate an “ethical eye” enabling healthcare professionals to navigate moral dilemmas with both analytical rigor and emotional awareness [16].

To address the need for a structured approach to ethical decision-making, we developed the **VIEW** (**V**isual **I**nsight for **E**thical **W**isdom) tool, a novel visual tool designed to enhance ethical decision making through reflective practices.

## Goal of Innovation

The VIEW tool integrates virtue-based and principle-based ethics by fostering reflective practices through visual mapping. Its name highlights its function of providing a clearer “view” of ethical dilemmas. By incorporating emotions, narratives, and structured ethical frameworks, VIEW offers an interactive and introspective approach for both educational and clinical contexts.

## Steps Taken for Development and Implementation

The VIEW tool was initially designed for a workshop by two early career physicians in a leadership program. Its effectiveness led to its use in two subsequent workshops.

## Implementation Steps

The VIEW tool employs a three-step process to guide users through ethical dilemmas: structured observation, emotional reflection, and narrative integration.

**Figure 1:**
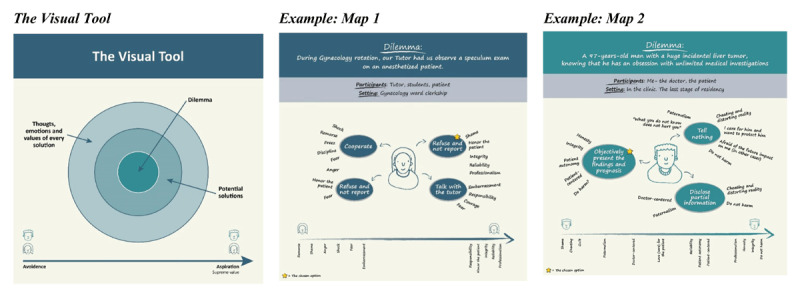
The Visual Tool.

The workshops followed a structured sequence using the visual tool (Figure 1), as follows:

1. **Graphic Mapping:** Using four concentric layers, participants created a map, i.e., a visual representation of an ethical dilemma. The outer layer documented a specific ethical dilemma from their professional practice, while the central layer contained the core moral question. The two layers in between mapped potential resolution pathways, with participants identifying each solution’s emotional responses and underlying values. This method helped participants systematically assess how different solutions align with their emotions and values.
2. **Instinctual Choices and Value Exploration:** Participants identified their instinctual choices among the potential solutions, and explored the underlying emotions and values driving the different options.
3. **Values Ranking:** Participants ranked values from avoidance to aspiration, fostering a multi-dimensional perspective of their relationship with each value and encouraging non-linear exploration of ethical decision making.

**Group Discussion and Reflection:** Participants shared their maps and reflections in a group setting, discussing their decisions’ emotional and long-term implications.

Workshops

To evaluate the tool, data were collected from three workshops:

1. May 2021: Ten junior family physicians (FPs).
2. February 2022: Fifteen family medicine educators.
3. July 2022: Six senior medical educators involved in ethical committees.

The Supplementary Table presents the demographic data of the workshop participants. Most participants had minimal exposure prior to ethics training, primarily limited to traditional case-based discussions during residency.

## Data Collection and Analysis

Data from the workshops were analyzed through an iterative process involving observations, visual maps, and semi-structured interviews. Two independent researchers manually coded and analyzed all data, refining themes through discussion. This rigorous qualitative approach ensured comprehensive interpretation of the participants’ experiences and insights.

***Observations:*** We took detailed field notes during the workshops, with observations focusing on participants’ interactions with the tool, group dynamics, and the nature of discussions. Verbal and non-verbal behaviors were documented, including collaborative problem-solving processes. These notes were coded, focusing on behaviors and discussions, capturing the reflections and evolving interpretations [17].

***Visual Maps:*** In total, 31 visual maps were generated by workshop participants during the sessions. Each map represents a participant’s articulation of an ethical dilemma, prioritized values, and emotional responses. The analysis process involved identifying recurring patterns and cross-referencing these patterns with the observations and interview data to ensure triangulation and comprehensive understanding of the themes.

***Interviews:*** We conducted four semi-structured interviews with workshop participants from diverse backgrounds within two weeks of the workshops to capture immediate reflections. Transcriptions were manually coded to identify recurring themes and patterns. The number of interviews was intentionally limited so as to complement insights from the observations and visual maps rather than to achieve theoretical saturation [18].

## Outcomes of the Innovation

We evaluated the VIEW tool’s effectiveness as a teaching method for enhancing ethical decision making among healthcare professionals. Workshop participants had diverse backgrounds and professional experience, and included early career and senior FPs, educators, and ethics committee members (See Supplementary Table).

The analysis of 31 maps demonstrated that the tool effectively addressed diverse ethical challenges. They ranged from complex end-of-life care decisions to everyday, yet ethically significant situations, such as sick leave certification.

Participants explored ethical dilemmas through personal real-life clinical examples. These personal scenarios sparked discussion and mapping exercises, fostering a deeper understanding of ethical reasoning processes. The analysis identified three primary themes: **interpretation** of dilemmas, **emotional responses**, and **values** in ethical decision making (See Table 1). For example, participants navigated conflicts between patient autonomy and professional integrity, balanced emotional distress with legal and ethical obligations, and reflected on their evolving PI.

**Table 1:** Examples of the Map Scenarios.


DILEMMA	PARTICIPANTS	SETTING	POTENTIAL SOLUTIONS	THOUGHTS AND EMOTIONS	VALUES

Presenting a 97-year-old man with a huge incidental liver tumor, knowing that he has an obsession with unlimited medical investigations (Figure 1)	Me- the doctor, the patient	Last stage of residency	Not to tell Tell partially and offer not to treat To objectively present the finding and prognosis, the clarification options	Cheating and distorting reality. I care for him and want to protect him. ‘*What you do not know does not hurt you*.’ Afraid of the future impact in other cases.	Paternalism, integrity, moral degradation Do not harm

During gynecology rotation, our tutor had us observe a speculum exam on an anesthetized patient (Figure 1)	Tutor, students, patient.	Gynecology clerkship	Collaboration refuse and do not report Report the tutor Talk to the tutor	Regret, shame, shock, helplessness, anger, responsibility, fear, embarrassment. Respect the patient.	Helplessness, remorse for reliability and professional integrity. Values at the center: reliability, professionalism, integrity

Discussing the euthanasia of a hospice patient with the patient and his family	Me- the doctor The patient The patient’s wife (primary caregiver), his children	At his home, near his bed. Three days after treatment ended	Give optimal palliative care, including sedation; Give life-ending care. Consult with a senior	The ‘right’ legal action; I am not ‘appreciated’ enough. Hospice values are ‘sacred’; Did he have time to say goodbye? Do not cause death to a conscious person. Patient suffering	Integrity Law vs. Ethics Autonomy Patient-centered Human dignity Do good, not harm; Sanctity of life

A dialysis patient is not cooperating and insults the staff, who feel threatened. The staff requests to stop treating him.	Patient, dialysis institute staff, Head of the department	Dialysis institute, during COVID-19	Support and back up the team and continue care. Terminate treatment at the institute. Continue with the routine without taking a stand.	The staff is entitled to respect and security. Who has priority? It is better to avoid decisions until things get better.	Patient-centered Teamwork Respect Responsibility for employees Object to violence Team security

Gynecological surgery, a woman in a lithotomy position. A janitor inappropriately touches the patient. I reported the incident to my supervisor, who warned me about the potential repercussions.	The patient, me, the janitor, and the operating room staff	Fifth-year student. Potential for residency in this ward. Operating room.	Tell the patient. Report to the department head/hospital manager/dean of clinical studies. Keep quiet. Confront the janitor. Contact the media.	Fear Not to report- What does that say about me? Feeling exposed, righteous I bypassed my superiors Fear of the janitor’s reaction I will not be able to specialize in the ward. Colleague hostility	Responsibility, Patient respect and dignity. Loyalty to patient vs. the staff. Integrity Do no harm Prevent sexual abuse (in the future)


***Case Example:*** A medical student recounts a gynecology clerkship incident two years ago. During a gynecology rotation, students were instructed to observe a speculum exam on an anesthetized patient, leading to a significant ethical dilemma (see Figure 1).

***Interpretation:*** The student faced conflicting values, particularly the tension between respecting the patient’s dignity and complying with the tutor’s instructions. Core values such as patient autonomy, consent, and professional integrity were at stake, as the student questioned the ethical propriety of observing the procedure without the patient’s explicit consent.

***Values:*** The student faced varying values, with patient dignity and autonomy at the aspiration end, emphasizing respect for consent and ethical responsibility. In contrast, authority acceptance and obedience are at the avoidance end, representing discomfort in following the tutor’s instructions. Values like professional integrity and clinical learning balanced the need for experience with ethical practice.

***Emotions*** such as regret, shame, and fear surfaced as the student navigated the situation, highlighting the inner conflict and the depth of reflection required when balancing these intersecting values.

Table 2 presents the themes and quotes.

**Table 2:** Themes and Quotes.


THEME	*QUOTE*

Tool’s effectivity	*The workshop provided closure and facilitated personal growth*.

	*The experience was insightful. I observed my fellow participants’ moral challenges and motivations, underscoring the horror and entrapment. However, I was able to process the situation effectively*.

	*The opportunity to document my ordeal in a protected environment was challenging yet crucial and insightful*.

	*It made me reconsider the options and revealed additional choices I hadn’t initially recognized*.

	*I felt I had processed the case more effectively and could draw significant insights from the challenging experience for the future. Additionally, I felt more self-compassion*.

Reflective thoughts on personal growth and professional identity	*It highlighted the workshop’s pivotal role in fostering ethical reflection and self-awareness, which are crucial for making sound moral decisions*.

	*The workshop highlighted the parallel between my past feelings of distress and their reemergence years later, underscoring its importance in processing and understanding my experiences*.

	*I realized there’s no absolute truth; everything is a matter of perspective. It affects how I think and feel about my personal and professional life*.

	*What does this mean about me?*

	*How will this affect my work in the future?*

	*Reflecting on the workshop, I realized my decisions were often fear-driven, overlooking the potential benefits for myself and others. This self-awareness prompted me to question my motivation*.

Avoidance	*The easiest way is to approach my supervisor, ask her to confront the conflict*.

	*Let it go… Not to think about it*.

Emotions	*Feeling exposed, righteous, fear*

	*Fear of the janitor’s reaction*

	*I am not ‘appreciated’ enough*.

	*I was angry about the patient’s family; why did they put me in this situation?*

	*I was afraid of my colleague’s hostility*.

	*When unsure about the right choice, I consult a colleague; I try to avoid involving feelings in the decision*.

Values	*I’ve realized that values evolve, and flexibility is necessary. Life isn’t black and white. I’ve consciously acted against my moral code, recognizing the importance of awareness. I may cross the line, but it’s a conscious choice*.

	*Witness reliability and professional integrity values: reliability, professionalism, integrity*.

	*The patient’s benefit often takes precedence over an absolute value like integrity*.

	*We are dealing with unacceptable conduct, including unreliability/transparency/manipulations- a clash of values*.

	*Act against my beliefs and values – how will this affect my work in the future – maybe I will do it again? Moral deterioration*.

Integrity	*Values of reliability and professional integrity at the center*.

	*On the spectrum: ambitions – courage and integrity, standing by my principles until giving up and moving on*.

Patient’s autonomy	*He [the patient] has a right to his body*.

	*The patient’s dignity, the patient’s right to his life*.

Avoiding harm	*I will not expedite the death of a person*.

	*Can I let them starve?*

	*My values are against causing passive/active death*.

Doing good	*Give optimal palliative pain relief treatment (including sedation*).

	*What is in the patient’s best interest?*

	*How does the patient feel? Is he suffering?*

Teamwork	*Unpleasant, a fight, fear of harassment, feeling exposed, righteous, I am bypassing my superiors*

	*Anger towards the patient and* [operating room] *staff*.

	*The care team is entitled to respect and security. Who has priority?*


For example, a reflection on the case presented in Map 1, Figure 1:

*I felt shocked and ashamed; it didn’t seem right to perform such an exam without the patient’s consent*.

This case exemplifies the complex ethical landscape that medical trainees navigate. Conflicting interpretations and emotional distress were also observed in other cases (See Table 1), emphasizing the need for a decision-making framework that integrates thoughts, emotions, and values for practical ethical reasoning in medical education.

### Interpretation of Dilemmas

Participants’ reflections revealed the challenges of balancing competing values and legal obligations, often exposing tensions between patient-centered care and professional integrity. For instance, one participant struggled with the ethical complexity of providing life-ending care to a hospice patient. Her reflections highlighted the emotional weight of the decision and its broader implications for the patient’s dignity and relationships:

Did he [the patient] have time to say goodbye?

This same participant also wrestled with the fear of violating the principle of nonmaleficence, expressing concern about the irreversible consequences of his actions:

*Do not cause death to a conscious person*.

Another participant, who had to deal with a non-cooperative dialysis patient, found it challenging to prioritize team well-being and security over patient dignity, especially in what was a high-stress environment:

*The staff is entitled to respect and security. Who has priority?*

In a different scenario, where inappropriate behavior by a colleague was observed, a participant expressed fear and doubt:

*What does that say about me? I bypassed my superiors… I will not be able to specialize in the ward*

Following their interpretation, participants’ real-time reactions to the dilemmas ranged from avoidance to confrontation, where avoidance often reflects the desire to escape emotional challenges:

*Let it go… Don’t think about it*.

Others, however, perceived avoidance in a positive light, as constructive collaboration:

*I will approach my supervisor and ask her to confront the conflict*.

### Emotional Responses

The emotional landscape revealed through the VIEW tool was rich and complex, with participants expressing a broad spectrum of emotions, including helplessness, anger, fear, regret, shame, and empathy, all closely linked to the ethical dilemmas they faced.

For instance:

*Reflecting on the workshop, I realized my decisions were often fear-driven, overlooking potential benefits*.

*I was angry with the patient’s family; why did they put me in this situation?*

The VIEW tool assisted participants in processing and identifying their emotional responses, allowing them to confront these feelings both intellectually and emotionally.

### Values in Ethical Decision Making

Participants articulated various values that guided their ethical decision making. Senior professionals struggled to rank emotional and value-based components, while less experienced participants found the process more intuitive.

As one senior participant noted:

*When unsure, I consult with a colleague and avoid involving feelings*.

In contrast, a younger participant reflected:

*Values evolve; flexibility is necessary. Life isn’t black and white*.

Values expressed during the workshops often aligned with the four principles of medical ethics, in addition to integrity and teamwork. For example:

Collision of values: ‘*The patient’s benefit often takes precedence over absolute values like integrity*.’

Patient autonomy: ‘*The patient’s dignity and right to his life*.’

Avoiding harm: ‘*I will not expedite the death of a person*.’

Doing good: ‘*What is in the patient’s best interest*.’

Integrity: ‘*Acting against my beliefs and values may lead to moral deterioration*.’

Teamwork: ‘*I am afraid of my colleague’s hostility…*’

The diversity in values enriched the learning experience and highlighted the importance of tools that accommodate varied ethical perspectives.

These insights were pivotal for fostering ethical reasoning, self-awareness, and reflective thoughts concerning PI, as expressed by the following quotes:

*I realized there’s no absolute truth; everything is a matter of perspective*.

*…the workshop’s pivotal role in fostering ethical reflection and self-awareness, which are crucial for making sound moral decisions*.

*I felt I had processed the case more effectively and could draw significant insights from the challenging experience for the future*.

Participants actively engaged with the tool, emphasizing personal values and offering a nuanced understanding of the complex interplay between individual and contextual factors in ethical decision making.

## Critical Reflection on the Process

Unlike increasingly AI-driven decision-support tools, our visual tool, the VIEW, offers a personalized and intuitive platform, providing a novel framework for ethical analysis by linking emotions, narratives, and structured decision-making. Its focus on integrating these dimensions encourages a holistic approach to navigating moral dilemmas, ensuring its relevance in both educational and clinical settings.

Our findings highlight differences in ethical reasoning based on professional experience. Senior physicians rely more on protocols and experience, while younger physicians often react emotionally. This emphasizes the need for tailored ethics education that integrates reflection to cultivate practical wisdom.

The VIEW complements established ethical frameworks such as the four principles model [8] and the four topics model [13], by incorporating integrity and teamwork, emphasizing their importance in collaborative, patient-centered care. It emphasizes the emotional and value-based responses of ethical analysis, allowing participants to reconcile instinctual virtue-based responses with principle-based reasoning. This fosters a more integrative and nuanced approach to ethical dilemmas.

The VIEW tool promotes a reflective practice by integrating ethical frameworks. It helps healthcare professionals strengthen their moral compass, self-awareness, and professional identity, engaging them more effectively than traditional methods like case discussions and lectures [19,20].

The tool effectively explores emotional and value-based dimensions of ethical dilemmas through personal examples, but it lacks real-time clinical scenarios. While this foundational approach aids in ethical reasoning, future research is needed to evaluate its applicability in dynamic real-world contexts as well as its long-term impact on medical practice compared with traditional ethics teaching methods. Nonetheless, participants reported improved clarity and resolution in their ethical decision making, suggesting the VIEW has the potential to enhance reflective practices and strengthen ethical reasoning in medical education.

This study has limitations, including a small sample size and the potential influence of cultural context, which may affect the generalizability of our findings. The qualitative approach introduces subjectivity, and the tool’s effectiveness across different specialties and experience levels remains unexplored. Further research in diverse settings is necessary to validate and refine the tool.

### Summary

The VIEW tool is an effective teaching method for fostering comprehensive reflective perspectives and deepening self-awareness. Its simple intuitive design helps align ethical analysis with personal values, supporting the development of a nuanced ethical framework and professional identity. Further research is recommended to validate VIEW applicability across diverse professional disciplines.

## Additional File

The additional file for this article can be found as follows:

10.5334/pme.1543.s1 Supplement Table. Participants’ demographics.
