# Supplementary material for: ‘As You Set Out for Ithaca’: VIEW- A Visual Tool for Teaching Ethical Decision Making in Medical Practice
Source: Perspect Med Educ. 2025 Jul 24;14(1):427–35. doi: 10.5334/pme.1543 (PMC12292043; doi:10.5334/pme.1543)
Supplement: Supplement Table. — Participants’ demographics. [file pme-14-1-1543-s1.pdf]

Supplement Table: Participants' demographics

Workshop No.	Setting	No. of Participants	Specialty and Seniority
1	Medical leadership training program	10	7 FM residents<br>3 young board-certificated FPs
2	Conference for Education in FM	15	12 senior FPs<br>3 social workers
3	National Medical Education Conference	7	Senior physicians from different specialties and medical educators.

FM- Family Medicine; FPs- Family Physicians
